# Pediatric membranous nephropathy: In the novel antigens era

**DOI:** 10.3389/fimmu.2022.962502

**Published:** 2022-08-09

**Authors:** Guoping Huang, Fei Liu, Ling Yu, Jingjing Wang, Junyi Chen, Jianhua Mao

**Affiliations:** Department of Nephrology, The Children’s Hospital, Zhejiang University School of Medicine, National Clinical Research Center For Child Health, Hangzhou, China

**Keywords:** pediatric membranous nephropathy, antigen, autoimmunity, PLA2R, Sema3B

## Abstract

Membranous nephropathy (MN) falls within the scope of a glomerular disease. MN exhibits subepithelial immune- complex deposition and capillary wall thickening which could occur in all age groups. In comparison with adult patients with MN, MN in pediatric population has a lower incidence and more secondary factors (e.g., systemic lupus erythematosus, infection, malignancy, or drug toxicity). Two target antigens for the immune complexes, PLA2R (identified in 2009) and THSD7A (in 2014), found in previous studies and first presented in adult MN, are found in pediatric patients suffering from MN and their antibodies are now an effective tool for diagnosis and monitoring in children and adolescents. Several novel antigens have been identified (e.g., EXT1/EXT2, NELL1, Sema3B, PCDH7, HTRA1, and NCAM1) over the past few years. Each of them represents different clinical and pathologic findings. In-depth research should be conducted to gain insights into the outcomes and pathophysiology of the above novel antigen-associated MN. Targeted treatment opinions for different novel antigen-related MN are under development both in adults and pediatric patients.

## Introduction

Membranous nephropathy (MN), characterized by capillary wall thickening and subepithelial immune-complex deposition, most commonly causes nephrotic syndrome among adults worldwide. MN has been rarely diagnosed among children (1, 2). Among all of them, 80% are with no secondary causes which are called primary MN (pMN). The rest 20%, which are called secondary MN (sMN), are associated with other diseases (e.g., an autoimmune disease, drug toxicity, malignancy, or infection) (1, 3). Due to the use of laser microdissection and tandem mass spectrometry techniques (MS/MS), several novel antigens for MN have been identified over the past decade. Our understanding of MN made a tremendous progress in pathogenesis, clinical classification, monitoring and treatment recommendations (4).

Exact data regarding the prevalence of MN remains unclear since studies with large population have been rare worldwide, especially data on pediatric patients. Men are more likely to develop MN than women, by a ratio of 2:1 (5); and it is diagnosed at a mean age of 50-60 years old (6). In comparison with adults, MN is uncommon in pediatric population(about 0.1 cases per 100,000 per year) and is mainly secondary to certain diseases (7). It accounts for <7% of all the pediatric kidney biopsies (8–10). Several considerable studies worldwide have suggested that the prevalence of MN is highly different between children<12 years old and adolescents. A retrospective analysis of 583 patients with onset ≤18 years of age in Pakistan reported a higher prevalence of MN in adolescents (18.5%) compared with the rate of patients ≤ 12 years old (3%) (11). A similar result was achieved by another national, cross-sectional biopsy survey including 71,151 patients in China, including 9% in adolescents while 3% in children <12 years old (9). Nevertheless, the frequency of MN is likely to be underestimated among children aged less than 10–12 years since children of this age group suffering from nephrotic-range proteinuria have been commonly treated with glucocorticoids empirically with no renal biopsy. Compared with adults, no specific gender distribution has been found in pediatric patients at the ratio of boy to girl between 3:1 and 1:1 in various studies (12, 13). Furthermore, the median age at presentation has shown a wide range between 7 and 15 years (12–14).

During the past two decades, there has been a noteworthy trend in some regions. The proportion of pediatric MN increased significantly from 3% in the first period (2004–2007) to 7% in the last period(2012-2014) reported from a 11-year national study in China (9), which was much higher than that in western countries (15). In addition, other major glomerulopathies still have stable proportions. Another earlier study also showed similar increased frequency in China, a retrospective investigation of 6049 patients receiving renal biopsy was made in a 10-year renal biopsy study conducted by a single Chinese nephrology center. Impacted by an increase in young patients with pMN (14–44 years old), the proportion of pMN at all ages rose from 16.8% between 2003 and 2007 (period 1) to 29.35% between 2008 and 2012 (period 2) (16), similar to the data acquired from other developing nations, including Turkey (17), Pakistan and India (18). We are not sure whether the incidence of MN has really increased significantly in the above regions or because of the increase of kidney biopsies in pediatric patients in developing countries due to improved economic levels and health care over the years. But researchers have suggested some possible mechanisms (18). The factors related to the environment are likely to increase the genetic propensity for pMN within the contaminated areas, the respective increase of 10 μg/m3 in PM2.5 concentration had a correlated with the increase of odds by 14% for pMN in the regions with PM2.5 higher than 70 μg/m3. However, more studies on the effects of genes and environmental factors on MN and other possible reasons for the increase are needed.

## PLA2R-associated MN

In 1959, researchers first identified an autoimmune response to an antigen as the cause of MN in the rat model (19). In the following decades, despite all the efforts, we didn’t find any novel antigens. Until 2009 M-type phospholipase A2 receptor 1 (PLA2R) was discovered as a target antigen in pMN (20). PLA2R refers to a transmembrane glycoprotein abundantly expressed by the human podocyte, present at the level of the foot process and on the apical surface, where it could be excreted into the urine as a vesicular structure in the course of disease (20, 21). It is the leading targeted auto-antigen in pMN (about 70%-80%), while its circulating autoantibodies are not found in normal controls or other glomerular diseases (20). The above characteristics laid the foundation for its later important role in the diagnosis, classification and therapeutic monitoring of pMN although its exact function is unclear. To date, the only experimental model of PLA2R-related MN is the mouse antigen (22), which is beneficial to gain insights into the molecular mechanism. An N-terminal cysteine-rich (CysR) domain, a fibronectin 2 domain and eight C-type lectin-like domains (CTLD) comprise the extracellular domain of PLA2R. The above domains contain different humoral epitopes. The CysR domain is identified as an immunodominant epitope (23). Some patients have CysR antibodies only. Other patients, however, develop epitope spreading and produce antibodies to the distal regions of the extracellular domain [e.g., CTLD8, CTLD7, and CTLD1 (24)]. Patients with only CysR antibodies were thought to be more prone to spontaneous remission and have a better prognosis (25). However, Linda Reinhard et al. have challenged this view (24). According to their research, total anti-PLA2R antibody levels, but not the PLA2R “epitope spreading”, are associated with the clinical outcome of MN patients. The quantification of PLA2R antibody titers has been used as a vital tool to verify the effectiveness of immunotherapy for PLA2R-related MN; it decreased following immunosuppressive treatment in parallel with clinical response (26).

After years of development, there have been several assays available to detect PLA2R antibodies. ELISA and the indirect immunofluorescence assay (IFA) are two of the most common assays. The second one is slightly more sensitive than the first one, whereas it is semi-quantitative, which means it cannot enable quantitative assessment (27). The manufacturer Euroimmun suggested that ELISA titers below 14 RU/ml are considered negative. However, researchers considered that it is more appropriate to set the cut-off as <2 RU/ml, which can reduce the false-negative rate (28). Both of them are less sensitive than western blot analysis and antigen staining of the kidney biopsy sample (29). The combination of IFA and ELISA is also a way to increase sensitivity. Besides the above assays, a novel addressable laser bead immunoassay has been developed, whereas it has not been used clinically (30).

Researchers worldwide have performed a new classification of patients with MN, PLA2R-positive and PLA2R-negative since the discovery of PLA2R in 2009. They have investigated the proportion of PLA2R-positive patients, their immunohistochemical and serological characteristics. Although PLA2R was initially considered the major target of pMN in adults, subsequent studies have indicated positive staining for it in pediatric patients. L. Nicholas Cossey et al. firstly identified PLA2R-positive on tissue staining in 10 of 22 pediatric patients with MN, providing a sensitivity of 45%. According to their research, PLA2R staining sensitivity in pediatric population is much lower than that in adults. They are likely to have a more diverse set of etiologies (31). A retrospective study conducted by Chinese experts, involving 187 adult pMN patients and 38 pediatric pMN patients, found positive PLA2R staining in 82.7% and 42.1% of adult and pediatric patients. While the adolescents exhibited similar clinical features and positive rate of PLA2R staining in comparison with adult patients, with a higher rate than young pediatric patients (32). A retrospective study between 2014 and 2017 was conducted in India, including 184 adults and 32 pediatric patients. 25 of the 32 pediatric patients aged between 6 to 17 years were pMN, and 7 were sMN. 11 of the 25 pMN (44%) were PLA2R-positive on tissue staining while serum was available for testing in 5 of them, 4 of which were positive (also positive on tissue staining) (33). None of the secondary MN were positive. Another Indian observational cohort study of adolescents with pMN found that 83% were PLA2R positive, either glomerular staining for PLA2R positive (14/18) or anti-PLA2R antibody positive (13/18) (34).

## THSD7A-associated MN

Time to 2014, Thrombospondin type 1 domain-containing 7A (THSD7A) was identified as a target antigen in adult pMN. It is a multidomain transmembrane glycoprotein expressed by the podocyte, serving as an auto-antigen in 2–3% of patients with MN (35). It is now recognized as a conserved basal component of the podocyte, localizing directly between the slit diaphragm and the GBM (36). From the perspective of structure, it also covers an extended extracellular domain and multiple epitodes along it. Its function and immunodominant epitope have been rarely studied. However, it seems that auto-antibodies target multiple regions of the protein (37) and maybe PLA2R and THSD7A even share a short common epitope in their N-terminal domain (38). The above finding breaks down the previously belief that individuals with pMN are autoimmune to one of the two proteins. In comparison with PLA2R, THSD7A-related MN patients are fewer, especially in pediatric population. Some of them are associated with malignancy (39). Furthermore, the level of THSD7A antibodies is associated with clinical remission, often decreasing with immunotherapy. In the retrospective study conducted in India with 184 adults and 32 pediatric patients, THSD7A was tested for in 29 pediatric cases and was positive in only one (a 17 years old pMN patient) (33).

## Novel antigen-associated MN

From 2018, several new and putative antigens have been discovered through laser microdissection and tandem mass spectrometry (MS/MS) (3). The basic premise in finding a novel antigen has been to identify a unique protein with high spectral counts in PLA2R-negative MN that is absent (or present in low spectral counts) in PLA2R-positive MN, and in control patients. Next, immunohistochemistry (IHC) was performed with the use of an antibody to the unique protein, and researchers found the presence of the membranous staining pattern along the GBM. Subsequently, the antigen and IgG were colocalized through confocal microscopy, and performed Western blot analysis on serum samples to detect circulating antibodies to the novel “antigen” of the new candidate antigens. This ushers a new era. To be specific, Sema3B appears to be most significantly correlated with pediatric MN. Sema3B-related MN is largely present in the pediatric population, especially in children<2 years old. It accounts for 1%-3% of all MN and 15% of pediatric cases. Antibodies to Sema3B can be detected under reducing conditions in Sema3B-related MN. Semaphorins are a group of secreted and transmembrane/membrane-bound proteins containing a conserved extracellular semaphorin (sema) domain of about 500 amino acids that is characterized by highly conserved cysteine residues (40). While Sema3B is a secreted protein which includes a sema domain, a plexin-sema-integrin domain, an Ig domain, and a basic domain. The sema 3 family have been detected in endothelial cells, podocytes, and tubular epithelial cells (41). Although the role played by Sema3B in the kidney remains unclear, it is considered to play an essential role in apoptosis and inhibition of angiogenesis. Existing studies have suggested that Sema3B was down-regulated in tumor tissues of patients with hepatocellular carcinoma and exerts anti-motility and anti-invasion effects on tumor cells (42). Cohort studies of Sema3B-related MN have been scarce. Clinical response varies in the small series. Some cases respond spontaneously, while others require immunosuppressive therapy (calcineurin inhibitors, rituximab or cyclophosphamide)  (43). French doctors recently reported the first case (a 7-year-old boy) of early recurrence after transplantation of MN associated with Sema3B. Anti-Sema3B antibodies were identified at transplantation. After recurrence he was treated with rituximab, and the antibodies were not detected 40 days after rituximab. The above case provides strong evidence that the disease arises from Sema3B antibodies entering the graft from the recipient circulation and suggests that Sema3B antibody could be a biomarker in the monitoring of patients with MN (44).

Other novel and putative antigens consist of EXT1 and EXT2 discovered in 2019, NELL1 in 2020, as well as PCDH7, HTRA1 and NCAM1 in 2021. EXT1/ETX2 deposits were mostly detected in underlying autoimmune disease (e.g., SLE. EXT1/EXT2-MN) and was predominantly present in young women, with a mean age of 35.7(SD ± 13.4). Approximately 1/3 of patients with class V lupus nephritis are positive for EXT1/EXT2 and the same percentage of mixed class lupus nephropathy (class III/IV with class V). However, EXT1/EXT2 are still recognized as putative antigens since the circulating anti-EXT1/EXT2 antibodies could not be found. Possible reasons may be either the serum antibodies titer is low or the antibodies could not recognize the epitopes on recombinant EXT1/EXT2 proteins. EXT1/EXT2-positive lupus membranous nephritis patients show better clinical outcomes compared with negative ones (45). NELL1 is present in both pMN patients and sMN patients(patients with malignancy). The MN may even precede the detection of the malignancy. Patients with NELL1-related MN are predominantly elderly, with a median age of 63.1 years (SD ± 10.4). One of the features in pathology is the segmental GBM distribution of the immune deposits in some of the glomeruli. Antibodies to NELL1 can be detected (46). PCDH7-related MN patients have a similar mean age to Nell1-related ones(61 years, SD ±11.7), with more male affected (male/female ratio of 3:1). Complement activation is minimal or absent both on IF microscopy and mass spectrometry. Antibodies to PCDH7 can be detected. Some of the patients in Mayo cohort went into spontaneous remission without immunosuppressive treatment (47). In 2021 NCAM1 was identified as an antigen in pMN and membranous lupus nephritis (LMN). It is present in younger patients than those seen in PLA2R or THSD7A positive MN patients. NCAM1-related MN accounts for 7% of all LMN cases (48). HTRA1-related MN is found in the elderly, with a mean age of 67 years and HTRA1 antibody levels show a correlation with clinical activity (49). Recently, Miller et al. described the largest retrospective cohort study of pediatric non-lupus membranous patients to date (50). 41 patients (16 children and 25 adolescents) with the diagnosis of MN from January 1995 to September 2020 were reviewed and tissue staining for novel autoantigens were performed. Of all the patients 4 children and 15 adolescents showed positivity: PLA2R+/SEMA3B- (13), SEMA3B+/PLA2R+ (2), SEMA3B+/PLA2R- (1), NELL1(1), EXT2+ (2), and THSD7A (0). The results of this study (15 positive in 25 adolescents and 4 positive in 16 children) confirmed the findings of previous studies on the prevalence of pMN (9, 11). And this cohort also did not show a male predominance (18 males:23 females) which is different from adults (12, 13). The remaining 22 cases with negative autoantigen staining may be related to an undefined antigen or other factors (genetic of environmental risk factors). Another interesting finding was that a 1.5-year-old male and a 20-year-old female with positive staining for both PLA2R and Sema3B. They are the first cases with dual PLA2R and Sema3B positivity reported in pediatrics. It suggested that autoantibodies to pMN maybe not mutually exclusive. However, more research is required to elucidate the pathogenicity and the correlation between antibody titers and clinical course.

Kidney biopsy is the standard diagnostic method for MN. Early stages of MN may show normal-appearing GBMs, as suggested by the result of light microscope (51). Lately, basement membrane spikes and pinholes can be seen on silver methenamine and periodic acid-Schiff stains. In immunofluorescence, the deposits results in a fine granular pattern of IgG staining along the outer surfaces of the capillary walls, while electron-dense deposits in electron microscopy are easily been identified in the basement membrane beneath the podocytes (52). There are several features which could help us to distinguish between pMN and sMN, such as mesangial or endocapillary proliferation (53). Besides, staining for IgG1, IgG2, and IgG3 primarily deposit in class Vlupus nephritis, whereas IgG4 predominates in PLA2R-related and THSD7A-related pMN (54). The IgG subclasses associated with recently identified antigens in MN are mainly IgG1 (3). The presence of antigens such as PLA2R or THSD7A can diagnose PLA2R/THSD7A-related MN when the antibodies disappeared and the patient becomes seronegative. Besides, antibody serology may be falsely negative early in the disease course owing to the circulating antibodies bind to the target antigens on the podocyte and are rapidly cleared from the blood. Patients with initial seronegative but persistent proteinuria with positive glomerular antigen staining should have serial evaluation of the antibody titers (55). However, more studies should be conducted on the role in diagnosis and the histological characteristics of the other novel antigens since they have a short discovery time.

## Indications for renal biopsy in pediatric MN patients

Impacted by the cost and complications of renal biopsy (especially for pediatric patients), people have been developing an alternative for it. Given the high specificity of PLA2R antibodies in patients with MN and the fact that PLA2R antibodies have not been detected in other glomerular diseases or healthy people, deferral of a kidney biopsy has been suggested in patients with nephrotic syndrome and PLA2R antibodies. As revealed by a study by Shane A. Bobart et al., in patients with no evidence of secondary cause (such as malignancy, infection, drug toxicity or an autoimmune disease) or diabetes mellitus and preserved kidney function (eGFR >60 ml/min/1.73 m2), a positive serum PLA2R antibody titer is equal to a 100% probability of diagnosing MN. It could be a reliable non-invasive method for the diagnosis of pMN (56). The above method is supported by the KDIGO 2021 guideline for the management of glomerular diseases (57). However, in comparison with adults, pediatric MN patients are somewhat different. Clinically, children with a diagnosis of nephrotic syndrome will be treated with steroids therapy first, and renal biopsy will be considered when urine protein does not turn negative after 4 weeks. In accordance with the recommendations of the KDIGO 2021 guideline (57), non-invasive PLA2R antibody testing may be performed in pediatric SRNS patients before a renal biopsy when secondary causes or persistent abnormal renal function are ruled out. When the patient has the positive PLA2R antibody serology, deferral of a renal biopsy is suggested. Further treatment can be guided by the antibody level.

## New classification of MN

The discovery of novel antigens in MN during the last decade has challenged the traditional classification of pMN and sMN. Because the above novel antigens could present both in pMN and be associated with a secondary disease. The pathophysiology, clinical features, laboratory findings, pathological biopsy characteristics and effect of treatment differ in each specific antigen-related MN (3). Sanjeev Sethi et al. argue that MN is just a pattern of glomerular injury, and different antigen-related MN are different diseases that have a common “membranous” pattern of injury. For PLA2R-related MN patients, we can guide treatment and observe response to treatment depending on the antibody titers. It is a good example. However, the possibility whether a similar method can be used for other antigen-related MN requires extensive research (3). Until then, researchers have proposed a new classification of MN based on detected antigens. The new classification consists of currently undetected antigens and other associated diseases. For instance, the EXT1/EXT2-related MN with class V lupus nephritis, MN with undetermined antigen associated with no associated diseases (58). In the novel antigen era, the traditional classification of pMN and sMN have been gradually abandoned.

## Treatment of MN

In accordance with the KDIGO 2021 guideline, adult patients with MN are divided into 4 different risk levels based on clinical and laboratory criteria, including low, moderate, high and very high risk. KDIGO recommends immunotherapy should be started in MN patients when there is at least one risk factor for disease progression (57). Risk-based initial treatment recommendations comprise rituximab for 6 months, cyclophosphamide + glucocorticoids for 6 months, calcineurin inhibitor ± prednisone, or calcineurin inhibitor + rituximab for ≥ 6 months. At 3 months or 6 months after diagnosis re-evaluation is important, as the changes in PLA2R antibody levels and clinical parameters may affect treatment indications. Supportive therapy (e.g., low-salt diet, rennin–angiotensin system, diuretics and prophylactic anticoagulation) takes on a critical significance, especially in patients with MN and proteinuria (57).

Nevertheless, impacted by gonadal suppression from alkylating agents, elevated risk of cancer and infection, and myelotoxicity, CNIs and/or rituximab are increasingly recommended for first-line treatment. Notably, the effect of rituximab in patients with MN was investigated in 4 RCTs worldwide. In the GEMRITUX trial at 31 French hospitals, the remission rate was 66% in patients treated with rituximab (total dose 750 mg/m2) and 45% in those who received conservative treatment after 23 months of follow-up (59). In the MENTOR trial in North America, involving 130 patients at the high risk of progressive disease, more patients receiving rituximab maintained remission at 12 months after therapy withdrawal (60% versus 20%) (60) though rituximab (total dose of 4 g) was not inferior to cyclosporine in inducing remission at 12 months after the treatment started. According to the STARMEN trial, the single dose of rituximab could prevent relapse after tacrolimus withdrawal though sequential therapy with rituximab (single dose 375 mg/m2) and tacrolimus (for 6 months) was inferior to the combination of cyclophosphamide and prednisolone (84% versus 58% at 24 months) in leading to remission (61). Rituximab (total dose 2g) was compared with cyclical cyclophosphamide and corticosteroids in a recent RI-CYCLO trial. The study had some limitations though it has been summarized that there was no signal of more benefit or less harm associated with rituximab versus a cyclic corticosteroid-cyclophosphamide regimen in the treatment of MN. Most patients covered in the study were at moderate risk with low anti-PLA2R antibody levels, and the study duration may have been not sufficient to capture the long-term toxicity potentially. A large, global, noninferiority trial for a more objective result should be conducted for a head-to-head pragmatic comparison (62).

Spontaneous remission is common in pediatric patients and renal function is always normal at presentation; in comparison with adults progressing to ESKD is rare in pediatric population. The most common clinical manifestation of MN in pediatric patients is nephrotic-range proteinuria (>50mg/kg) or the nephrotic syndrome (steroid-resistant nephrotic syndrome), and proteinuria combined with hematuria is more common compared to adults. Some severe cases may also present with impaired renal function (63). Chinese researchers first reported the long-term cumulative renal survival rates of ESKD in children with pMN were 95.3% (5-year) and 67.8% (10-year), respectively. They also reported that hypertension and proteinuria ≥ 50 mg/kg/day were associated with renal outcome in children with pMN (13). Because of the rarity of MN in the children and its good prognosis with no treatment, no randomized controlled studies have been conducted on MN treatment in pediatric patients. Primary forms of MN, especially in adolescents, could be treated similarly to adults. While children with MN should be managed in expert centers and individually, in which the adverse effects of immunotherapy are considered (57). Secondary forms of MN require management of the underlying condition. Literature about immunotherapy management in pediatric patients with MN is rare. Lee et al. (12) reported a clinical remission rate of 68% in 19 pediatric patients with MN receiving immunotherapy management; Ramachandran et al. (64) demonstrated in an uncontrolled study of 48 pediatric patients with MN that 2/3 cases responded to immunosuppressive therapy. Unlike adult MN patients, renal biopsy is not usually performed in pediatric population with steroid-sensitive NS. The degree of pediatric MN responding to steroid monotherapy remains not clear. As revealed by the review of the adult MN literature, steroids alone are not beneficial in comparison with conservative therapy in pMN (65). As reported by several case series, pediatric MN is likely to respond to corticosteroids. In a retrospective review of 12 pediatric pMN patients, 50% of the cases do not respond to steroids, and there was complete or partial response in the rest (14); Another retrospective study conducted in Pakistan including 75 pediatric patients who underwent renal biopsy showed that 11 had MN. Of the 11 patients, 6 were steroid sensitive, 2 were steroid dependent, and 3 were steroid resistant (66). For other immunosuppressants, Valentini et al. (14) demonstrated a 75% response to a 3-month course of oral cyclophosphamide therapy (2 mg/kg/day) with alternate-day steroids. Lee et al. (12) reported CNI use (cyclosporine) in 3 cases with 100% response within 6 months but relapsed after drug withdrawal. Chen et al. (10)reported cyclosporine use in 3 steroid-resistant patients and tacrolimus use in 2 cases with similar results. Bhimma et al. (67) reported 4 pediatric pMN patients treated with mycophenolate mofetil (1200 mg/m^2^ per day) with partial remission at 6 months (with low dose steroids and ACE inhibitors). Experience of immunosuppression management in pediatric patients is limited in few uncontrolled case series to date. It remains a big challenge for pediatric nephrologists worldwide to choose one immunosuppressant over another preferentially.

Several small, uncontrolled case series and case reports provide insights into treatment and prognosis of pediatric patients with MN. In a single-center prospective study including 48 pediatric pMN patients in India, 98% patients received immunosuppressive therapy: mycophenolate mofetil (2.1%), azathioprine (4.3%), prednisolone alone (4.3%), rituximab (14.9%), CNI/GC (21.3%) and cyclical CYC/GC (53.1%). It showed two-thirds patients responded to immunotherapy. And among all of the patients 60% patients treated with rituximab, it is useful as a first-line and rescue therapy (64). Two cases of adolescent MN with elevated anti-PLA2R levels and nephrotic-range proteinuria who did not achieve remission with steroids were reported successfully treated with rituximab (68). It was reported that a 4-year-old patient with THSD7A-related MN was well treated with rituximab (69). Although there has been no clear recommendation regarding the use of rituximab as a first-line treatment for pediatric patients with MN, the growing number of case series and case reports suggest us rituximab is a promising agent to manage pMN. Nevertheless, the optimal dosing and timing of rituximab in pediatric patients have not been established. Some patients received 1 g rituximab (days 0 and 15), some received 375 mg/m2 (×4 weekly doses), and others underwent CD19-targeted rituximab therapy (64). Although rituximab is effective, the side effects in pediatric patients should be considered, including severe infections, neutropenia and hypogammaglobulinemia. They should be significantly monitored as any immunosuppressive agent.

**Figure 1:**
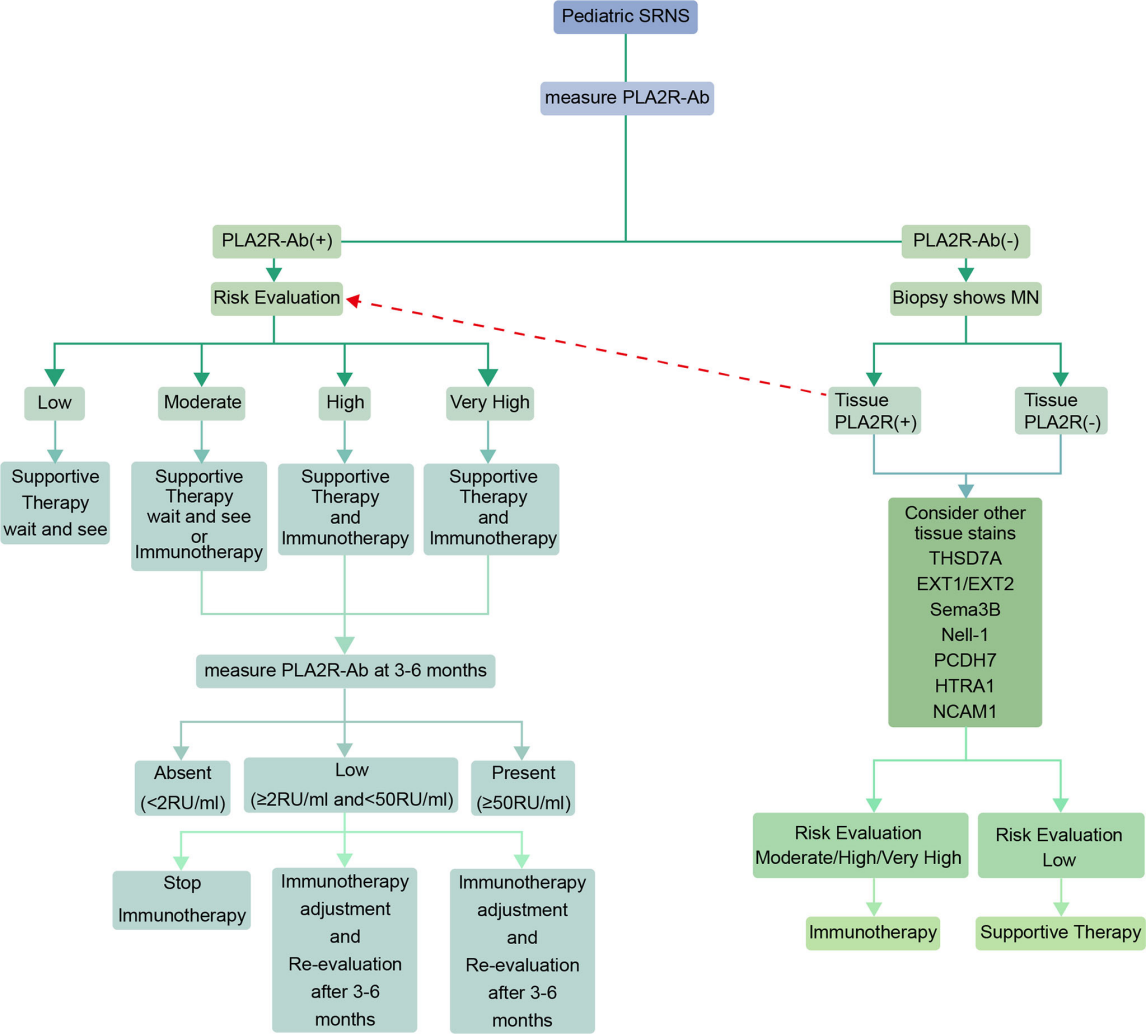
Algorithm for the diagnosis and treatment advice in pediatric MN patients (In cases of pediatric steroid-resistant nephrotic syndrome). The risk evaluation can be based on recommendations in the 2021 KDIGO guidelines for adult MN (56). The interval between PL2AR antibody levels measurement is generally 3-6 months, and it should be shortened for pediatric patients with high baseline levels. (Patients with initial positive PLA2R staining and positive antibodies in sera whose antibodies turn to negative spontaneously or following immunotherapy should be in the low risk category. Patients who have not undergone treatment or have relapsed after long-term complete remission may be in the moderate/high/very high risk categories when the tests show positive for PLA2R staining but negative for antibodies in sera. PLA2R antibodies are sometimes undetected because the buffer capacity of the kidney is not exceeded) (70).

In the novel antigens era, when a pediatric patient is diagnosed with steroid-resistant nephrotic syndrome (SRNS), he/she may first be measured for PLA2Rab and if positive then a risk evaluation can be performed to develop a treatment plan by referring to the 2021 KDIGO guideline. Subsequently, PLA2Rab level should be monitored regularly, and the next immunotherapy plan should be determined in accordance with the antibody level (The interval is usually 3-6 months, and the interval between tests should be shortened for those with high antibody levels at baseline). If negative for PLA2R antibodies, a renal biopsy and kidney tissue staining will be required. The treatment plan is based on kidney tissue staining (**Figure 1**). Generally speaking, there is no consensus on whether immunosuppressive therapy should be used in pediatric patients with MN who have partial response after 4 weeks of steroids use; Menon et al. suggested that management of MN in pediatric population should evaluate the risks and benefits of immunosuppressive therapy. The conservative treatment may be used instead of immunosuppressive therapy in patients with normal renal function and sub-nephrotic proteinuria (7). But many researchers hold other opinions. In a prospective study mentioned above including 48 children and adolescents with pMN, 44 of whom presented with nephrotic syndrome and the remainder with non-nephrotic proteinuria. 47 (97.91%) cases received immunosuppressive therapy, and over two-thirds of the patients respond to immunosuppressive therapy (However, it is unknown whether immunosuppressive agents were used in the 4 cases with non-nephrotic proteinuria). And the authors suggested that the choice of immunosuppressive therapy should be based on the patients’ characteristics and nephrologists’ experience (64). In a retrospective study including 37 children with MN (all of them have received a full dose of steroids for up to six weeks), 19 of whom were given other immunosuppressive agents after the renal biopsy, 7 continued steroids-alone therapy, and only 11 received no immunosuppressive therapy. The proportion of children receiving immunosuppressive therapy was 70% (however, we still do not know how many cases were with partial remission before renal biopsy) (71). Therefore, through these two studies we can infer that most pediatric nephrologists prefer immunosuppressive therapy for children and adolescents with MN. However, these data are derived from a small number of case reports and uncontrolled case series. More studies are needed in the future.

## Discussion

Novel antigens (EXT1/EXT2, NELL1, Sema3B, PCDH7, HTRA1, and NCAM1) in MN have been found over the past few years using MS/MS. Since they have not been well studied, it is still controversial whether they are true antigens or just biomarkers. Further studies should be conducted on the pathogenic role of antibodies, including the correlation of antibody levels with the disease process and recurrence after transplantation. Animal models also need to be established. Only a few cases regarding early recurrence after kidney transplantation and parallel results of immune and clinical activity have been reported thus far (43, 47, 49). For pediatric nephrologists, studies on Sema3B take on a greater clinical significance, including the localization of Sema3B to the surface of podocytes and the determination of the specificity of circulating antibodies to glomerular Sema3B. In the future, the new technologies (MS/MS, multiplexed immunofluorescence imaging technology, single-cell RNA sequencing) will be beneficial to identify additional targeted antigens in all the forms of MN and provide a better understanding of the similarities and differences between different antigen-related MN (72).

Clinicians have insufficient experience in the treatment of pediatric MN patients because there are no RCT studies to date. Adolescent patients are primarily referred to adult standards, while younger children are treated in accordance with the experience of different doctors in different medical centers, which brings a great challenge to pediatric nephrologists. The treatment of pediatric patients with MN should be more individualized and targeted, especially considering the side effects of immunotherapy. The use of second- and third-generation CD20 antibodies [e.g., ofatumumab, Obinutuzumab, and ocrelizumab (73–76)] could be tried, especially in certain refractory membranous nephropathies or in pediatric patients who develop serum sickness after the use of rituximab.

Belimumab, a human IgG1λ monoclonal antibody which inhibits BAFF, can affect B cell proliferation and survival. It could be used in combination with rituximab in patients with MN. In a small open-label, prospective, single-arm study it had some effect (77). The involvement of CD19–CD20-CD38+CD138+ long-lived memory plasma cells in bone marrow and kidney may be conducive to the low level of sustained complete remission in some patients with MN who treated with rituximab. Immunoadsorption and plasmapheresis have been employed in some patients to improve effects (78). More studies should be conducted on the above new therapies.

There has been a rapid progress in the diagnosis and treatment of adult patients with MN over the past decade because of the discovery of PLA2R. A question is raised that whether other novel antigens identified in the last few years can guide the treatment of MN in pediatric population in the future, as well as whether there are any targeted treatment options for different novel antigen-related MN. We believe they will be the focus of research in the next few years.

## Author contributions

GH and FL are joint first authors. They were responsible for literature search and article writing. JW and LY drew the chart. JC modified the format of the article. JM revised the article. All authors contributed to the article and approved the submitted version.

## Conflict of interest

The authors declare that the research was conducted in the absence of any commercial or financial relationships that could be construed as a potential conflict of interest.

## Publisher’s note

All claims expressed in this article are solely those of the authors and do not necessarily represent those of their affiliated organizations, or those of the publisher, the editors and the reviewers. Any product that may be evaluated in this article, or claim that may be made by its manufacturer, is not guaranteed or endorsed by the publisher.

## Abbreviations

MN, membranous nephropathy; pMN, primary membranous nephropathy; sMN, secondary membranous nephropathy; MS/MS, laser microdissection and tandem mass spectrometry; ELISA, Enzyme-Linked Immunosorbent Assays; IFA, indirect mmunofluorescence assay; GBM, Glomerular basement membrane; IHC, immunohistochemistry; LMN, lupus membranous nephropathy; SRNS, steroid-resistant nephrotic syndrome; CNI, calcineurin inhibitor; RCT, Randomized Controlled Trial; ESKD, end stage kidney disease; GC, glucocorticoid; CYC, cyclophosphamide.
